# Continuous Dihydrolevoglucosenone Recovery Using Commercial Membrane Technology

**DOI:** 10.1021/acs.oprd.4c00494

**Published:** 2025-04-09

**Authors:** Andreas Dejaegere, Alessandro Napoli, Thomas S.A. Heugebaert, Christian V. Stevens

**Affiliations:** SynBioC Research Group, Department of Green Chemistry and Technology, Faculty of Bioscience Engineering, Ghent University, Ghent 9000, Belgium

**Keywords:** dihydrolevoglucosenone, waste reduction, continuous solvent recovery, Zaiput extraction technology

## Abstract

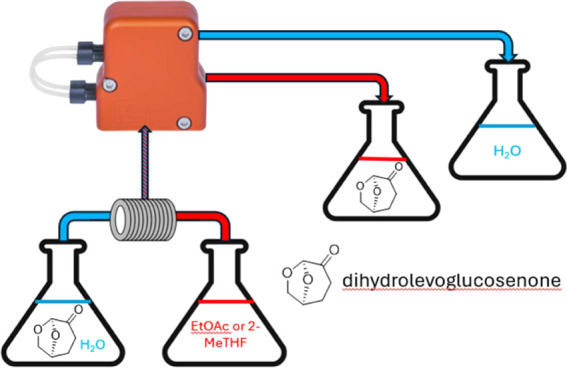

Cyrene, or dihydrolevoglucosenone (DHL), is a biobased and biodegradable solvent that can be produced in two steps from cellulose. It has properties similar to dipolar aprotic solvents such as NMP and DMF, both of which raise significant concerns regarding environmental and human health. As such, dihydrolevoglucosenone offers a promising alternative. The use of this sustainable solvent enhances the environmental profile of chemical reactions. However, target compounds synthesized in dihydrolevoglucosenone are mainly purified using an aqueous workup, leading mostly to the disposal of DHL in an aqueous waste stream. This study focuses on recovering dihydrolevoglucosenone through back-extraction processes.

## Introduction

Dipolar aprotic solvents such as DMSO, DMF, DMAc, and NMP are extensively utilized in organic synthesis and pharmaceutical research due to their high polarity, high solubilizing power, stability and versatility in facilitating various chemical reactions. They play an important role in the synthesis of active pharmaceutical ingredients (APIs). However, solvents like DMF, DMAc and NMP are considered undesirable based on green solvent guidelines,^1^ because of their detrimental effects on human health and hazards to the natural environment, significant wastewater generation and high-energy-input requirements for the recovery and waste disposal.^2^ Dimethylformamide (DMF), for example, has been linked to serious health issues, such as liver damage and reproductive toxicity in case of prolonged exposure. Therefore, it is classified as a hazardous substance by several regulatory bodies.^3^ In the EU, the use of DMF has been restricted since 2023 because of the reproductive health hazard. DMAc and NMP are also of high concern due to their reproductive toxicity and their potential to harm the unborn child.^4^ An additional problem is the growing demand for these solvents in the pharmaceutical industry and other chemical processes.^2^ It is therefore clear that the search for safer and more sustainable alternatives is urgently needed. Minimizing and avoiding the use of such solvents has become one of the most important facets of green chemistry. In recent years, significant efforts have been made to address this challenge, leading to the development of novel and environmentally sustainable reaction systems and solvents that share key characteristics with traditional dipolar aprotic solvents, such as high polarity, excellent solubilizing power, stability, and versatility.^2^

**Figure 1:**
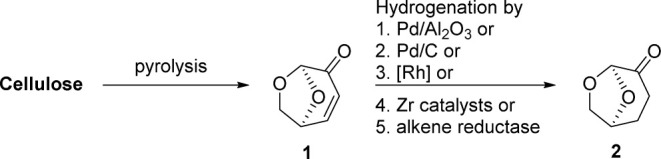
Two-step production of dihydrolevoglucosenone from cellulose.^9^

Dihydrolevoglucosenone is an example of such an alternative solvent that can address these challenges. This sustainable dipolar aprotic solvent is produced in two steps from cellulosic biomass (Figure 1). In the first step, cellulose is pyrolyzed, producing levoglucosenone (LGO) **1**. Over the years, various methods using different acids have been employed to achieve the highest possible yields. In 2011, Circa Group reported and patented a thermal process that produces LGO in 40% yield, involving the catalytic pyrolysis of biomass with phosphoric acid and sulfolane.^5^ Achieving high-purity LGO involves multiple distillation steps, which are essential as the purity of dihydrolevoglucosenone largely depends on the quality of the separated LGO. This is due to the high selectivity (99%) of the subsequent hydrogenation process that converts LGO to DHL.^6^

Levoglucosenone is hydrogenated in a second step to yield dihydrolevoglucosenone (DHL) **2** (Figure 1). Various catalysts, such as Pd/Al_2_O_3_,^7^ Pd/C,^2,7^ rhodium-based and Zr catalysts,^6^ are commonly used for this hydrogenation step. In addition to the chemical catalytic hydrogenation processes, an enzymatic method was recently reported that uses an alkene reductase.^8^

The similarity in polarity and solvency properties make dihydrolevoglucosenone a good candidate for the replacement of for example DMF.^9^ Dihydrolevoglucosenone does not contain nitrogen and sulfur heteroatoms in its molecular structure as typically found in polar aprotic solvents. The absence of these heteroatoms is beneficial as they are known to lead to atmospheric pollution when the solvent is incinerated.^10^ In contrast to DMF, DMA, and NMP, dihydrolevoglucosenone is free from systemic toxicity, reproductive toxicity, and skin irritation, positioning it as a more environmentally friendly option. Dihydrolevoglucosenone is also readily biodegradable, breaking down 99% within 14 days through aerobic biological processes.^11^ This biodegradability is significant because it reduces the risk of environmental accumulation, unlike traditional petrochemical solvents, which can persist and cause harm to ecosystems. The structure of DHL, being derived from renewable cellulose, allows microorganisms to metabolize it efficiently, minimizing long-term pollution risks. Thanks to its greener and safer character, dihydrolevoglucosenone offers a more sustainable and environmentally friendly profile.

Since the discovery of DHL, several applications have been explored.^9^ It was found to be an efficient medium for several C–C bond forming reactions,^12,13^ nucleophilic substitutions,^14^ synthesis of substituted amides and ureas,^15,16^ for a range of polymerizations.^17^

Dihydrolevoglucosenone, though a sustainable alternative to conventional dipolar aprotic solvents, has certain limitations that need to be considered in organic synthesis. It is incompatible with strong oxidizing or reducing agents and with strong acids or bases, particularly in processes involving heat. Furthermore, its high viscosity can create challenges for efficient mass transfer during reactions.^11^ This challenge can be addressed by using vigorous stirring to promote thorough mixing of the reaction mixture.^18^

However, despite its promising characteristics and green character, dihydrolevoglucosenone is often discarded after use rather than being recovered or recycled. This practice undermines its full potential as a sustainable solution, as the environmental benefits are not fully realized when it is not reclaimed. Efforts to improve its recovery and reuse are necessary to enhance its role as a truly circular and eco-friendly solvent. Unlike DMF, dihydrolevoglucosenone is more challenging to remove through standard distillation due to its high boiling point of 227 °C.^19^

For certain reactions in dihydrolevoglucosenone purification of the desired product can be simplified by adding water to the reaction mixture. In such situations, DHL and water-soluble byproducts produced during the reaction are transferred to the aqueous phase, often resulting in a relatively pure product without the need for complex purification methods.^16^ However, in current practice, the resulting aqueous phase containing dihydrolevoglucosenone and byproducts is often discarded, without DHL being recovered.

To address this challenge, we explored a back extraction process using an organic solvent to recover dihydrolevoglucosenone. The goal was to separate DHL from the aqueous phase with the help of a second, easily removable and recyclable organic solvent, preferably one adhering to the green solvent selection guidelines to minimize environmental and health impacts. Different types of extraction separators, including liquid–liquid (L/L) and liquid–gas (L/G) separators, are commonly used for solvent recovery and reuse. This study specifically focuses on recovering dihydrolevoglucosenone from an aqueous phase using a liquid–liquid separator.

## Choice of the Extraction Solvents

For the extraction experiments, two solvents were selected: ethyl acetate (EtOAc) and 2-methyltetrahydrofuran (2-MeTHF). These are both solvents that are classified as acceptable green solvents.^1^

Ethyl acetate is considered considerably green due to its low toxicity, biodegradability and potential for being sourced from renewable materials such as bioethanol and biobased acetic acid.^20^ These attributes make it a safer and more sustainable option compared to traditional petrochemical-derived solvents. Additionally, it can be recovered and reused in various industrial processes, reducing waste and promoting circular use. However, its high volatility contributes to the emission of volatile organic compounds (VOCs). These can affect air quality and contribute to pollution. Despite this drawback, ethyl acetate remains a preferable solvent in green chemistry practices due to its balance between effectiveness and environmental impact.

2-Methyltetrahydrofuran is considered a green solvent due to several key attributes. First, it can be synthesized from renewable feedstocks such as agricultural waste or biomass, particularly from furfural, making it a more sustainable alternative to traditional petrochemical-derived solvents such as tetrahydrofuran (THF). Additionally, it has a lower toxicity compared to many conventional solvents, enhancing safety for both workers and for the environment. Its biodegradable nature leads to a more facile breakdown in the environment, further minimizing its ecological impact. THF, on the other hand, is sourced from petrochemicals and is classified as “not readily biodegradable”.^21^ Moreover, 2-MeTHF exhibits favorable solvent properties compared to THF, including a reduced tendency to form explosive peroxides and a lower vapor pressure, which decreases emissions of volatile organic compounds (VOCs) that contribute to air pollution. Collectively, these characteristics position 2-MeTHF as a desirable choice in green chemistry, offering a balance between performance and environmental responsibility.^22^

## Continuous Extraction Systems

**Figure 2:**
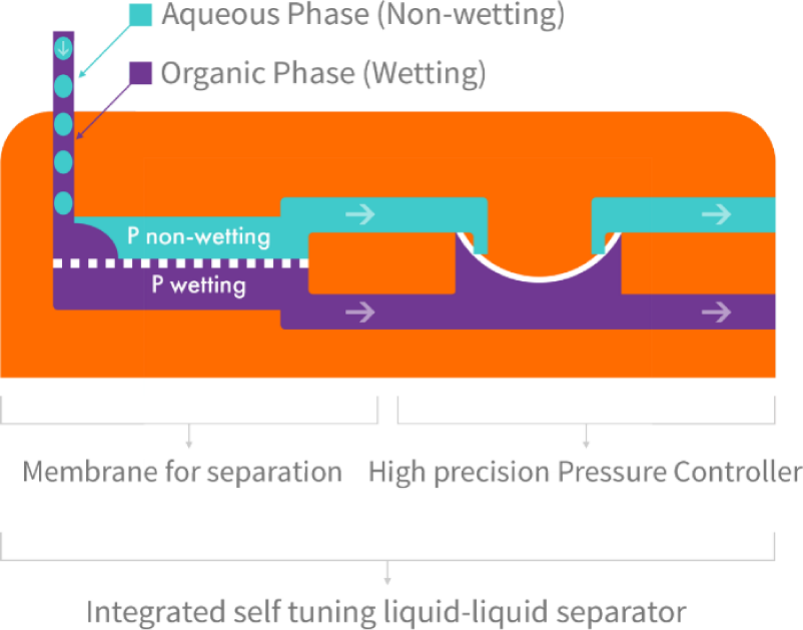
Concept of membrane separation used by Zaiput.^23^

The objective of this research was to evaluate a continuous extraction system for the recovery of dihydrolevoglucosenone from an aqueous medium. We started by investigating available technologies suitable for continuous extraction in a laboratory setting. Membrane separators from Zaiput Flow Technologies were identified as a promising solution due to their ability to efficiently separate organic and aqueous phases in a continuous flow setting. The Zaiput separator used in our research is specifically designed for laboratory-scale extractions, with the capability to handle incoming flow rates ranging from 0 to 10 mL/min. The Zaiput separator is equipped with a porous membrane, which can be either hydrophobic or hydrophilic, along with a pressure controller (Figure 2). This configuration enables the selective separation of two phases, such as aqueous and organic liquids. When the mixture enters the separator, the phase with an affinity for the membrane, known as the ″wetting″ phase, fills the membrane pores. In contrast, the ″nonwetting″ phase is repelled and does not penetrate the pores. Once the membrane is filled with the wetting phase, a pressure differential is applied, calibrated to allow only the wetting phase to pass through while blocking the nonwetting phase. The system maintains a consistent pressure differential across various flow rates and inlet conditions, making it a versatile, user-friendly modular unit ideal for plug-and-play applications.

## Experimental Work

For the experimental work of this article, dihydrolevoglucosenone was provided by Circa. Ethyl acetate and 2-methyltetrahydrofuran were purchased from Sigma-Aldrich (ACS reagent, ≥ 99.5%). For the batch experiments a regular separation funnel (Schott Duran) was used. For the continuous extractions, the phase separators (Zaiput SEP-10 and the Zaiput Multistage Separation MS-10) were purchased from Zaiput Flow Technologies. For the experiments with 1 separation unit, Vapourtec SF-10 peristaltic pumps were used to pump the 2 phases into the system. For the countercurrent extractions with the MS-10 platform, an Asia Syringe pump was used.

**Figure 3:**
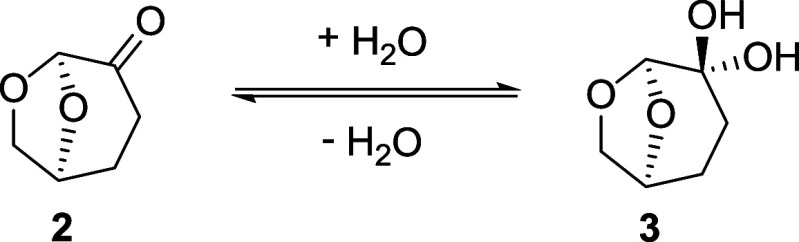
Equilibrium between dihydrolevoglucosenone **2** and its geminal diol **3** when dissolved in water.^24^

Initial experiments consisted of batch extractions, using a normal separation funnel. The aim of these was to get a first idea about the extraction equilibrium. For these experiments 10 mL dihydrolevoglucosenone was dissolved in 20 mL water. An excess amount of water is required to fully dissolve the DHL in the aqueous phase, as an equilibrium is formed between dihydrolevoglucosenone **2** and its geminal diol **3** when mixed with water (Figure 3). To fully shift this equilibrium toward the formation of the geminal diol, it is essential to use twice the volume of water in relation to DHL.^24,25^

The DHL/water mixture was extracted with an equal volume of ethyl acetate (30 mL). These experiments showed that the mixing of the two phases is crucial. The longer and more intense the mixing is, the higher the percentage of dihydrolevoglucosenone that can be extracted. In order to extract as much dihydrolevoglucosenone as possible, 5 consecutive extractions were performed. Fresh extraction solvent was used for each extraction. The results of these experiments are listed in Table 1. When using ethyl acetate, an extraction efficiency of 56% was obtained after 5 extractions with a mixing time of 1 min. When applying a mixing time of 5 min, an overall efficiency of 85% could be obtained. When repeating the same experiments with 2-methyltetrahydrofuran, only 64% extraction efficiency could be reached, both at a mixing time of 1 and 5 min.

**Table 1:** Overall Efficiencies Reached in Batch Extractions with EtOAc and 2-MeTHF

Mixing time	Extraction 1	Extraction 2	Extraction 3	Extraction 4	Extraction 5
1 min^a^	19%	33%	47%	52%	56%
5 min^a^	43%	62%	74%	82%	85%
1 min^b^	27%	40%	49%	59%	64%
5 min^b^	21%	39%	48%	59%	64%

a EtOAc was used as extraction solvent.

b 2-MeTHF was used as extraction solvent.

In a second step, continuous flow extractions were performed with 1 Zaiput separator unit. Again, 10 mL of dihydrolevoglucosenone was dissolved in double the volume of water and extracted with 30 mL of organic solvent (EtOAc or 2-MeTHF). In these experiments, 5 consecutive extractions, using the same separator unit, with fresh extraction solvent each time, were performed. For each extraction a flow rate of 1.5 mL/min was applied using a Vapourtec SF-10 pump. Since batch extractions have shown that intense mixing increases the efficiency, inline static mixers were placed inside the tubing before the separator unit. At room temperature, the extraction efficiencies were relatively low, with only 56% of the dihydrolevoglucosenone recovered after five extractions with EtOAc. Previous batch experiments demonstrated that a longer contact time is crucial for sufficient mixing between the two phases. However, in our continuous flow setup, extended contact times were not feasible, prompting the need to find an alternative solution to enhance mass transfer rates. Temperature plays a key role in influencing the partitioning behavior between the organic and aqueous phases.^26^ To investigate the effect of temperature on our system, the mixing zone of the flow setup was heated in a water bath. The results show that heating the mixing zone at 65 or 75 °C gives rise to a higher extraction efficiency (Table 2). In this system, the efficiency could be improved up to 92% when using EtOAc. In this experiment 0.58 g DHL/min could be recovered. Since 2-MeTHF has a higher boiling point compared to EtOAc, we could heat the mixing zone up to 75 °C. Under these conditions it was possible to extract almost 99% of the dihydrolevoglucosenone. In this experiment 0.62 g DHL/min could be recovered.

In the next step, countercurrent extraction was implemented. Compared to a cocurrent setup, countercurrent usually provides a theoretical higher extraction efficiency per stage. For these experiments the MS10 multistage extraction platform of Zaiput Flow Technologies was used.

**Figure 4:**
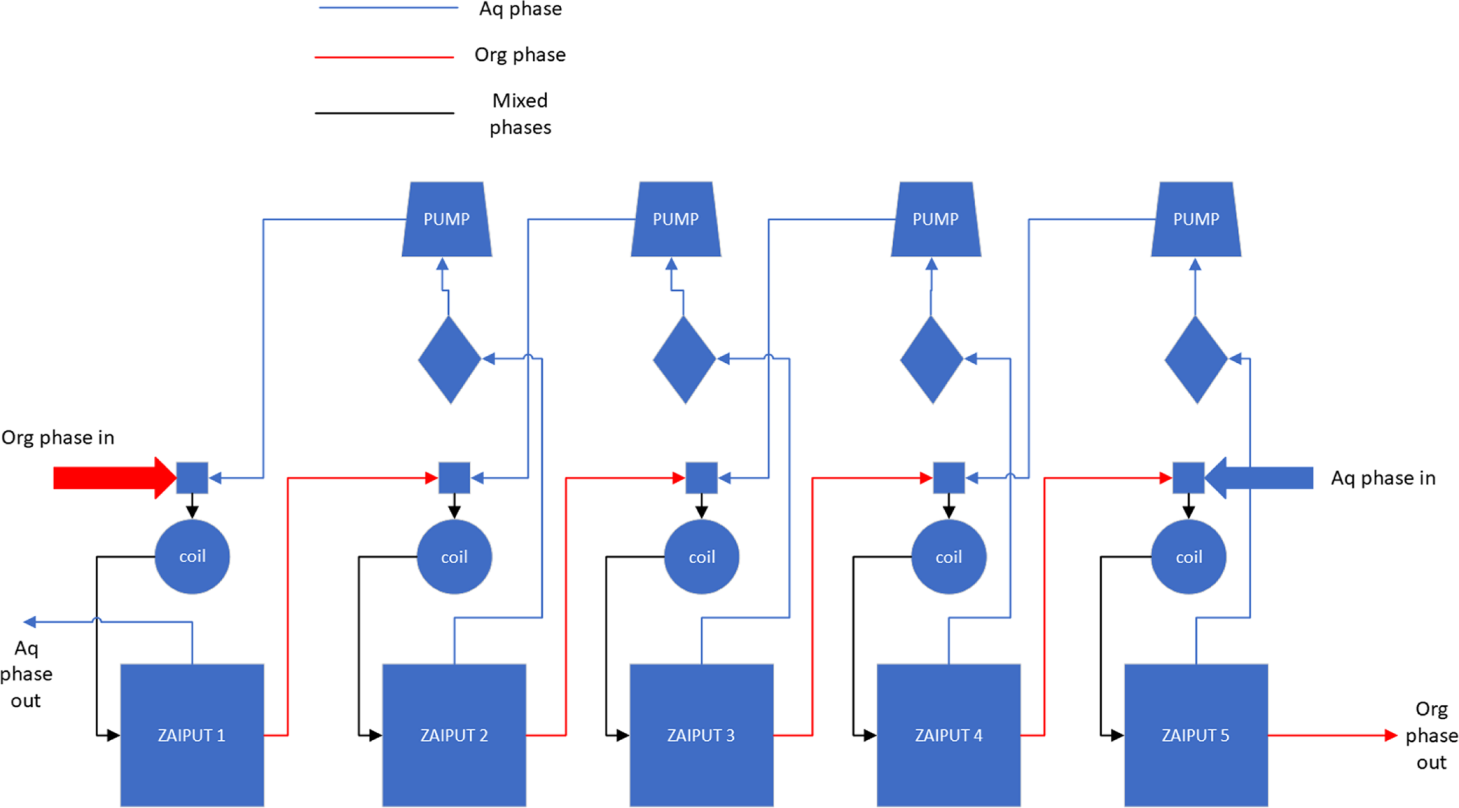
Schematic overview of the countercurrent extraction platform.

Five individual separators can be combined in a platform designed to perform continuous countercurrent extractions (Figure 4). In this system, the aqueous outlet from one extraction unit serves as the aqueous feed for the next unit, while the organic phase flows in the opposite direction. To promote effective mixing between the two phases before each separator, mixing coils are incorporated into the platform. These coils provide sufficient residence time for mass transfer between the two liquid phases, allowing equilibrium to be achieved and resulting in successful extraction. To maintain proper pressure and ensure continuous operation of the platform, interstage pumps and permeate flow sensors are installed. These components enable efficient separation and stable platform performance. The system (MS-10 extraction platform) used in this research is intended for laboratory-scale extractions and is capable of handling flow rates between 0 and 10 mL/min. Although the extraction efficiency at each individual stage may be modest, the overall system can achieve a significantly higher extraction yield.

For these experiments, 30 mL dihydrolevoglucosenone was dissolved in 60 mL water (again a 2/1 volume ratio). This mixture was extracted with 90 mL extraction solvent (EtOAc or 2-MeTHF). Both phases were pumped into the platform using the Asia Syringe pump at a flow rate of 1.5 mL/min. In this setup a maximum efficiency of around 55–60% could be reached when using EtOAc at room temperature (0.38 g DHL/min recovered). When the same experiment was performed with 2-MeTHF, an efficiency of 42% could be reached (0.26 g DHL/min recovered). A key feature of the platform is that it only accepts one aqueous and one organic phase, making it impossible to use fresh extraction solvent for each extraction stage. Therefore, the EtOAc and 2-MeTHF consumption is five times lower than in the previous extraction experiments.

In the final stage of our work, we used the countercurrent extraction system to recover dihydrolevoglucosenone after it had been used in a chemical reaction. The aim was to assess the purity of the recovered solvent, ensuring the effective removal of reaction byproducts and impurities. GC-MS and ^1^H NMR analysis were used to determine whether dihydrolevoglucosenone retained its integrity and remained suitable for reuse. These findings can provide valuable insight into the efficiency of the extraction system and its potential for sustainable solvent recovery.

**Figure 5:**
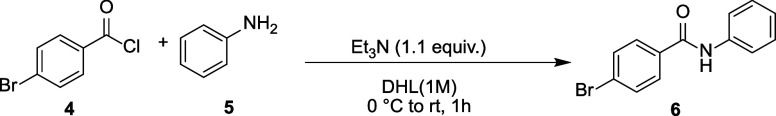
Synthesis of 4-bromo-*N*-phenylbenzamide **6** from 4-bromobenzoyl chloride **4** and aniline **5**.^16^

We selected the synthesis of 4-bromo-*N*-phenylbenzamide **6** from 4-bromobenzoyl chloride **4** and aniline **5** in dihydrolevoglucosenone as the model reaction (Figure 5).^16^ The detailed reaction procedure is provided in the Supporting Information.

The purification procedure for this reaction involves adding water to the reaction mixture to precipitate the product. This results in a water/dihydrolevoglucosenone mixture, which can then be purified using our extraction method.

The reaction was carried out on a 10-g scale to ensure sufficient dihydrolevoglucosenone was available for recovery using the countercurrent extraction platform. After the purification procedure of the reaction, we ended up with 50 mL dihydrolevoglucosenone dissolved in 150 mL water and this mixture was extracted with 200 mL EtOAc. Both phases were pumped into the extraction platform using the Asia Syringe pump at a flow rate of 1.5 mL/min. This extraction experiment had an efficiency 35% (0.17 g DHL/min recovered). The recovered dihydrolevoglucosenone was then analyzed using GC-MS. We analyzed samples of both fresh and recovered DHL, using dodecane as an internal standard in both cases. Our calculations determined that the recovered dihydrolevoglucosenone had a purity of 92%. The calculation method is detailed in the Supporting Information.

## Discussion

The results show that the back extraction of dihydrolevoglucosenone from an aqueous waste stream is possible with different extraction systems. When using the classical batch extraction, sufficient mixing time is crucial to reach an equilibrium between the two phases in order to extract more dihydrolevoglucosenone. We observed that a higher efficiency could be reached with EtOAc when increasing the mixing time for each extraction step. In the case of 2-MeTHF, no significant increase was observed. This difference can be explained by considering the water solubility and partition coefficient or log *P* value of the extraction solvent used. This value is a crucial factor when selecting a solvent for extracting a compound from an aqueous phase. This value represents the logarithm of the partition coefficient, which quantifies the distribution of a compound between an aqueous phase and an organic solvent.^27^ With a relatively high log *P* value of 1.1, 2-MeTHF is less hydrophilic and not miscible with water, resulting in lower mixing efficiency during extraction compared to ethyl acetate (EtOAc), which has a lower log *P* (0.71) and better water compatibility.^28^ The higher water solubility and moderate polarity of EtOAc facilitates a more effective extraction process.

The continuous extraction experiments with a single Zaiput separator show that it is possible to recover more than 90% of the dihydrolevoglucosenone that is used. In this setup it is possible to make use of both the mixing and temperature effects. By heating the mixing zone, the efficiency can be significantly increased. It is known that temperature has an influence on the partitioning behavior between an organic and aqueous phase.^26^ In that way it has an influence on the extraction equilibrium of the system. However, it is crucial to consider the boiling point of the extraction solvent. Once this temperature is exceeded, the solvent will begin to boil, leading to a failed extraction and subsequent separation process.

The MS10 multistage extraction platform used in the countercurrent experiments should theoretically provide better extraction efficiency. However, this platform suffers from the major design flaw that it, due to the built-in pumps, does not allow heating of the solvent mixture. As such, it was impossible to take advantage of the crucial temperature effect. As a result, only an extraction efficiency of 55–60% could be reached at room temperature. However, these experiments offer several important advantages. As this is equally efficient as the experiments with one Zaiput separator at room temperature (Table 2), the key difference is that in the case of five consecutive extractions, five times more extraction solvent is required compared to the MS10 countercurrent extraction platform. So this strategy allows a significant reduction in the costs associated with solvent use. Additionally, since the platform operates without the need to heat the incoming solvent mixture, energy costs are also minimized.

**Table 2:** Overall Efficiencies with 1 Zaiput Separator Using EtOAc and 2-MeTHF

Temperature	Extraction 1	Extraction 2	Extraction 3	Extraction 4	Extraction 5
25 °C^a^	19%	33%	47%	52%	56%
65 °C^a^	52%	75%	88%	91%	92%
75 °C^b^	54%	78%	90%	95%	99%

a EtOAc was used as extraction solvent.

b 2-MeTHF was used as extraction solvent.

To make this research attractive for the pharmaceutical industry, it is essential to address the scalability challenge. The membrane separators used in this research are designed for laboratory-scale applications. However, Zaiput Flow Technologies also offers membrane separation equipment suitable for pilot and production scales. For both individual separators and platforms, the pilot-scale systems can handle flow rates between 20 and 200 mL/min, while the production-scale system accommodates flow rates ranging from 200 to 3000 mL/min.^29,30^

**Figure 6:**
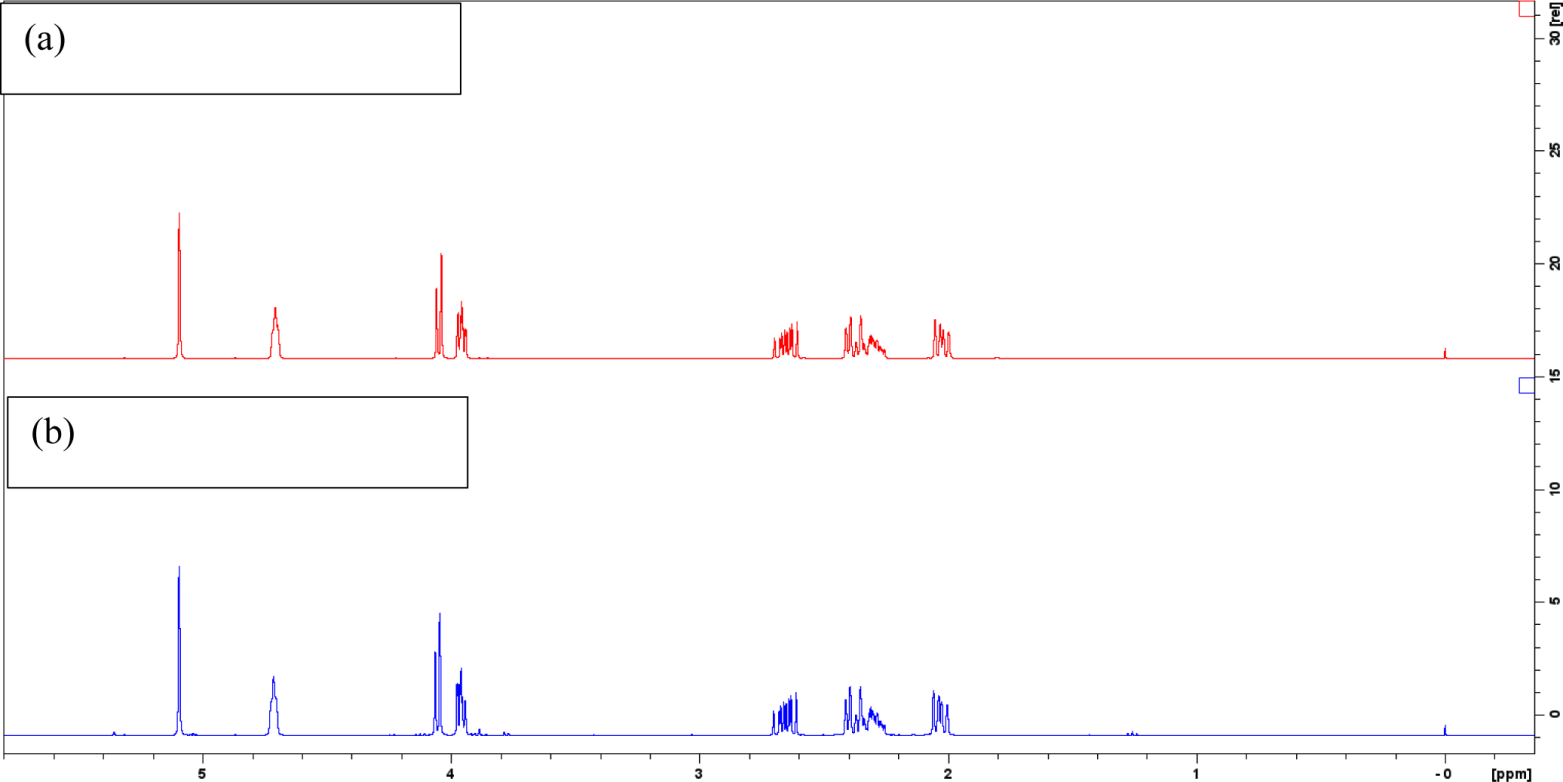
^1^H NMR spectrum of fresh dihydrolevoglucosenone (a) and recovered dihydrolevoglucosenone (b).

Another important consideration is the quality of the recovered dihydrolevoglucosenone. To ensure that the solvent can be reused, it should retain as many of its beneficial properties as possible. To achieve this, we can investigate the reversion of the geminal diol formation and any degradation that takes place during the extraction process. Using ^1^H NMR analysis, it can be confirmed that no geminal diol is present after evaporation of the organic extraction solvent. The ^1^H NMR spectrum of the recovered dihydrolevoglucosenone shows no visible degradation products when compared to the spectrum of fresh dihydrolevoglucosenone (Figure 6).

The quality and purity of the recovered dihydrolevoglucosenone become even more important after use in a real chemical reaction. Our analysis showed that the recovered DHL, used in the synthesis of 4-bromo-*N*-phenylbenzamide (6), had a purity of 92%. The required purity level depends on the intended reuse application, with additional purification steps often necessary to meet the standards. Depending on the carbon footprint of virgin solvent production, further distillation to pharmaceutical grade or redirection to other industries may be more suitable.^31^

## Conclusion

In conclusion, we have demonstrated that dihydrolevoglucosenone can be recovered from aqueous phases using various back extraction systems. In batch experiments, an overall efficiency of 85% was achieved after five consecutive extractions using EtOAc, while 64% was obtained with 2-MeTHF, provided the two phases were mixed for 5 min. In continuous flow extractions with a single Zaiput SEP-10 separator, an efficiency of 56% was obtained after five extractions at room temperature. By utilizing the beneficial effects of temperature in this setup, more than 90% of the dihydrolevoglucosenone was recovered. Countercurrent extraction experiments with the Zaiput MS-10 multistage platform, conducted at room temperature, yielded an efficiency between 55 and 60% when EtOAc was used and 42% in the case of 2-MeTHF. The key advantage of this method over previous continuous extractions is that it eliminates the need for heating and requires five times less extraction solvent, leading to reduced energy and solvent costs.

The use of commercial extraction systems like Zaiput provides opportunities for scaling up our extraction methods, as Zaiput offers systems for both pilot and production scales. Additional research is required to assess the efficiency and productivity of these systems.

Testing our extraction system with an actual reaction mixture demonstrated that the recovered dihydrolevoglucosenone has a high purity (>90%). Depending on the applications for which it will be reused, additional purification techniques can be applied.

It is clear that the extraction of dihydrolevoglucosenone with acceptable green organic solvents like EtOAc and 2-MeTHF creates opportunities for the use of dihydrolevoglucosenone as a biobased alternative solvent for DMF and other polar aprotic solvents. Dihydrolevoglucosenone recovery has the potential to improve the sustainability of an overall process and further lower the environmental footprint of certain reactions in the pharmaceutical industry.

## Supporting Information Available

General information about the experimental work and description of extraction experiments that were done (PDF)
