# Supplementary material for: Continuous Dihydrolevoglucosenone Recovery Using Commercial Membrane Technology
Source: Org Process Res Dev. 2025 Apr 9;29(4):1076–82. doi: 10.1021/acs.oprd.4c00494 (PMC12056688; doi:10.1021/acs.oprd.4c00494)
Supplement: Supplementary file 1 — op4c00494_si_001.pdf [file op4c00494_si_001.pdf]

Supporting Information

Continuous dihydrolevoglucosenone recovery using commercial membrane technology

Andreas Dejaegere,[a] *Alessandro Napoli,*[b] *Thomas S.A. Heugebaert,*[a] *Christian V. Stevens*[a*]

[a]SynBioC Research Group, Department of Sustainable Organic Chemistry and Technology, Faculty of Bioscience Engineering, Ghent University, Coupure Links 653, B-9000 Ghent, Belgium

[b]Circa Group AS, Karenslyst Allé 53, 0279 Oslo, Norway

*Email: Chris.Stevens@UGent.be

CONTENT

General information

Description of the experimental work

Calculation of the extraction yields

Figure S1: ^{1}H-NMR spectrum of dihydrolevoglucosenone

Synthesis of 4-bromo-*N*-phelybenzamide

GC-MS analysis

References

General information

For the experimental work of this article, dihydrolevoglucosenone (DHL) was provided by Circa. Ethyl acetate (EtOAc) and 2-methyltetrahydrofuran (2-MeTHF) were purchased from Sigma-Aldrich (ACS reagent, ≥99.5%). For the batch experiments a regular separation funnel (Schott Duran) was used. For the continuous extractions, the phase separators (Zaiput SEP-10 and the Zaiput Multistage Separation MS-10) were purchased from Zaiput Flow Technologies. For the experiments with 1 separation unit, Vapourtec SF-10 pumps were used to pump the 2 phases into the system. For the counter-current extractions with the MS-10 platform, an Asia Syringe pump was used.

NMR spectra were measured with a Bruker Avance Nanobay III NMR spectrometer. The components were dissolved in deuterated chloroform ($CDCl_3$) and tetramethylsilane (TMS) as an internal standard.

Experimental procedure

Batch extractions

For these experiments, 10 mL of dihydrolevoglucosenone was dissolved in 20 mL water (2/1 ratio). This mixture was extracted with 30 mL EtOAc or 2-MeTHF. For the extraction, a regular separation funnel (Schott Duran) was used. After phase separation, the organic phase was evaporated under reduced pressure to obtain the extracted dihydrolevoglucosenone. The aqueous phase was again extracted with 30 mL fresh EtOAc or 2-MeTHF. The extraction yield of each step were calculated using a mass balance of the system.

Continuous extraction with 1 Zaiput separator

10 mL of dihydrolevoglucosenone was dissolved in 20 mL of water (2/1 ratio) and extracted with 30 mL EtOAc or 2-MeTHF. Both phases were pumped into the system at a flow rate of 1.5 mL/min using a Vapourtec SF-10 peristaltic pump. In order to maximize the mixing of both phases, inline static mixers were placed inside the tubing before the Zaiput SEP-10 separator unit. After separation, the organic phase was evaporated under reduced pressure.

For the heated extraction, the mixing zone placed inside a warm water bath and heated up to 65 or 75 ˚C, depending on the extraction solvent that was used.

Continuous extraction with the MS10 multi-stage extraction platform

For these experiments, the MS-10 multi-stage extraction platform of Zaiput was used to perform counter-current extractions. The aqueous phase consisted of 30 mL of dihydrolevoglucosenone dissolved in 60 mL water. 90 mL of EtOAc or 2-MeTHF was used as organic phase for the extractions. Both phases were pumped into the platform using a Syris Asia piston pump at a flow rate of 1.5 mL/min. After separation, the organic fraction was evaporated under reduced pressure using a rotary evaporator.

Calculation of the extraction yields

The extraction yields of all the experiments were calculated by making the mass balance of dihydrolevoglucosenone inside the system. Knowing that dihydrolevoglucosenone has a density of 1.25 g/mL, the total extraction efficiency of each step could be calculated using the following formula:

$$Extraction\ efficiency = \frac{Mass\ DHL\ extracted}{Total\ mass\ DHL} \times 100$$

The total mass of dihydrolevoglucosenone is the amount of dihydrolevoglucosenone that was initially mixed with water.

Figure S1: ^{1}H-NMR spectrum of dihydrolevoglucosenone

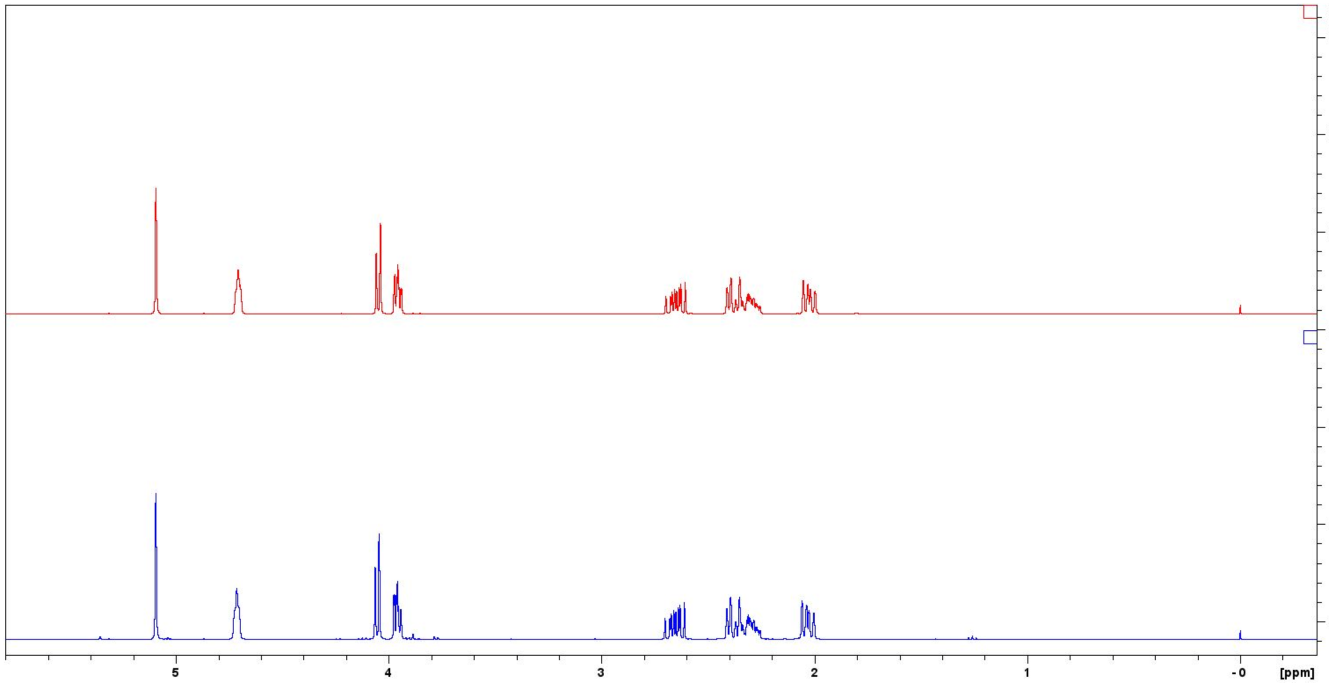

Synthesis of 4-bromo-*N*-phenylbenzamide

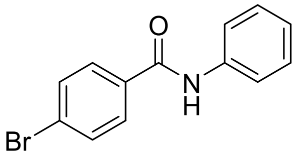

6

To a stirred solution of 4-bromobenzoyl chloride (10.973 g, 50 mmol) in dihydrolevoglucosenone (50 mL, 1 M) at 0 ˚C were added triethylamine (5.566 g, 55 mmol) and aniline (4.657 g, 50 mmol). The resultant mixture was allowed to warm to r.t. over 1 h. Water (100 mL) was added and the mixture was stirred for 1 h. The precipitate was filtered and washed with water. The residue was dissolved in EtOAc, dried over sodium sulfate and the solvent was removed under reduced pressure to give 4-bromo-*N*-phenylbenzamide (**6**, 10.92 g; 79%) as an off white solid.

Figure S2: ^{1}H-NMR spectrum of 4-bromo-*N*-phenylbenzamide

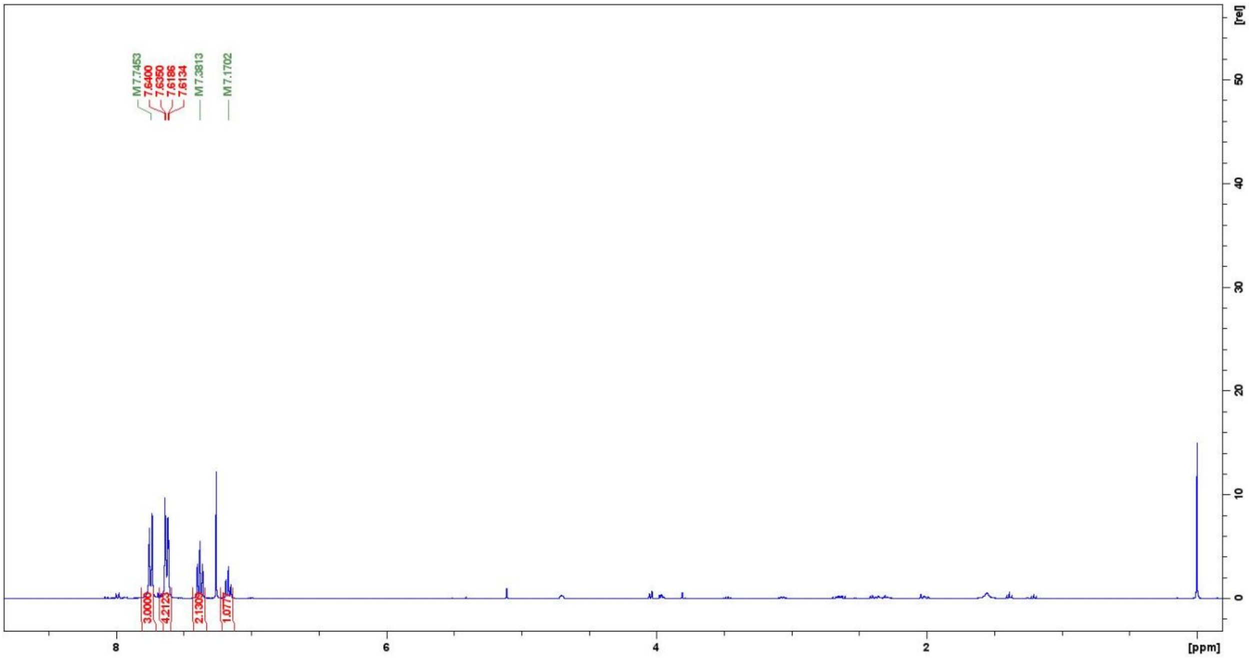

Detailed spectral data, including NMR, IR, and MS analyses, can be found in the original publication of Bousfield et al.[1]

GC-MS analysis

GC analysis was performed on an Agilent 6890N gas chromatograph equipped with an Agilent J&W HP-5 (30 m x 0.25 mm x 0.25 μm) column and FID detector. Dodecane was used as an internal standard to compare the samples and estimate the purity of the recovered dihydrolevoglucosenone. Using the Agilent Mass Hunter software, the peak areas were determined and the purity was calculated as follows.

$$Purity = \frac{(Peak\ area\ DHL\ recov.)/(Peak\ area\ IS\ recov.)}{(\text{Peak area DHL pure})/(\text{Peak area IS pure})}$$

Using this formula, a purity of 92% was determined.

Figure S3: GC-MS spectra of freshly and recovered dihydrolevoglucosenone

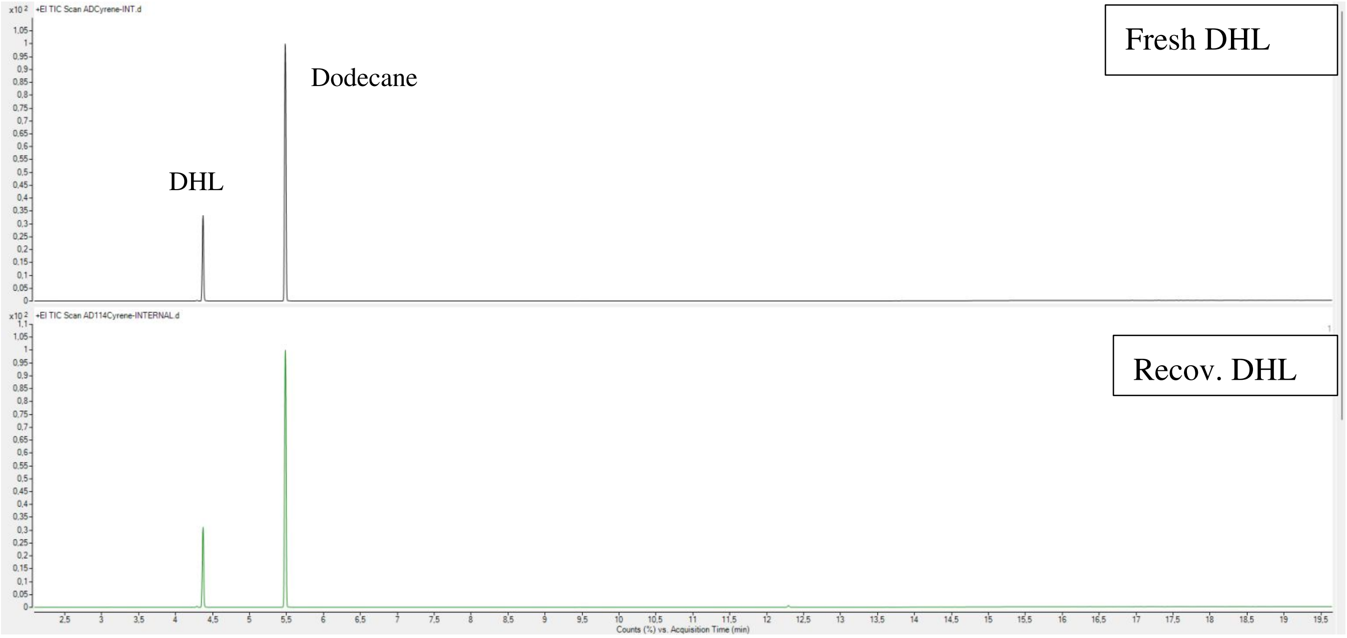

References

(1) Bousfield, T. W.; Pearce, K. P. R.; Nyamini, S. B.; Angelis-Dimakis, A.; Camp, J. E. Synthesis of Amides from Acid Chlorides and Amines in the Bio-Based Solvent Cyrene™. *Green Chemistry* 2019, *21* (13), 3675–3681. https://doi.org/10.1039/C9GC01180C.
